# Pain Perception and Stabilometric Parameters in People With Chronic Low Back Pain After a Pilates Exercise Program: A Randomized Controlled Trial

**DOI:** 10.1097/MD.0000000000002414

**Published:** 2016-01-15

**Authors:** Antonino Patti, Antonino Bianco, Antonio Paoli, Giuseppe Messina, Maria Alessandra Montalto, Marianna Bellafiore, Giuseppe Battaglia, Angelo Iovane, Antonio Palma

**Affiliations:** From the Sport and Exercise Sciences Research Unit, University of Palermo, Palermo, Italy (APatti, AB, GM, MAM, MB, GB, AI, APalma); Posturalab Research Institute, Palermo, Italy (APatti, GM); and Department of Biomedical Science, University of Padua, Padua, Italy (APaoli).

## Abstract

Various exercise interventions, such as Pilates exercises and traditional physical therapy methods, are employed to decrease low back pain (LBP). Nonspecific low back pain (NSLBP) is distinct from LBP, however, as the distribution of pain is restricted to the region between the costal margin and the inferior gluteal. The aim of our randomized controlled trial was to evaluate the effects of a program of Pilates exercises on pain perception and stabilometric parameters in patients with NSLBP.

Thirty-eight participants were randomly allocated, using a 1:1 scheme, to either the experimental group (EG) or control group (CG). The EG completed a 14-week program of Pilates exercises, performed thrice per week under the supervision of an exercise specialist, while the CG was managed with a social program only. Measures of posturography and Oswestry Disability Index (ODI) for pain perception were obtained at baseline (T^0^) and after the 14 weeks of intervention (T^1^).

Posturography measures improved for patients in the EG, with both eyes open and eyes closed (*P* < 0.05). There were no statistical differences in posturography in the CG. ODI decreased significantly in both groups over the 14 weeks of the study protocol: EG, T^0^, 13.7 ± 5.0 compared with T^1^, 6.5 ± 4.0 (*P* < 0.001); and CG, T^0^, 10.7 ± 7.8 compared with T^1^, 8.4 ± 7.8 (*P* < 0.01). A greater extent of reduction in pain was achieved in the EG.

The Pilates exercise program yielded improvements in pain and posturography outcomes. Our study also confirms the applicability of posturography in evaluating postural instability in patients with NSLBP. Due to our relatively small study group, future studies would be necessary to confirm our findings.

## INTRODUCTION

In Europe, from 20% to 30% of adults are affected by musculoskeletal conditions (MSCs) at least once in their lifetime.^1,2^ Low back pain (LBP) is the most prevalent MSC and 1 of the most common causes of disability. Nonspecific low back pain (NSLBP) is defined as pain that is localized below the costal margin and above the inferior gluteal.^3,4^ Many factors have been shown to contribute to acute episodes of NSLBP, including incorrect activation and force of the erector spine muscles, strength imbalance of the trunk muscles, and overall decrease in trunk muscle strength.^5^ Various approaches have been developed to manage LBP, with exercise prescription, including Pilates exercises. We recently published a systematic review providing evidence of the positive effects of Pilates exercise in the management of patients with LBP.^6^ The Pilates exercise method is defined as a technique that focuses on core stability, posture, breathing, flexibility, strength, and muscle control.^7^ The Pilates approach focuses on active awareness of the use of trunk muscles to stabilize the pelvic–lumbar region.^8^ There is evidence that lumbar stabilization exercises improve deep muscle strength that ultimately can improve the range of motion.^9^ It has been suggested that the Pilates method is more effective than a minimal physical exercise intervention in reducing pain and disability in the short term; however, it is unclear which factors of the exercise intervention and which type of exercises may be responsible for these improvements.^6^ The Pilates approach is unique in that during the exercise session, particular attention is drawn to the quality, precision, and control of movement, including specific attention to breathing and sensory feedback.^10^ There is increasing evidence of alterations in postural control and static balance in patients with LBP.^11^ The dynamic interaction of sensory input, central processing, and integration to motor output for the sensorimotor control of posture can be reliably measured with posturography,^12^ providing clinically relevant information on postural balance.^12^ In 2014, Bianco et al^13^ showed the utility of posturography for the identification of alterations in postural stability in patients with cervical trauma. In our study, we used a combination of measurement of pain perception and stabilometry in people with NSLBP to evaluate the effectiveness of a Pilates program of exercise in this clinical population. The specific aims of our randomized controlled trial (RCT) were, first, to evaluate the effects of a Pilates program of exercise on pain perception of people with NSLBP and, second, to evaluate the change in posturography measurements before and after a Pilates exercise program.

## MATERIALS AND METHODS

### Study Design and Context

**FIGURE 1:**
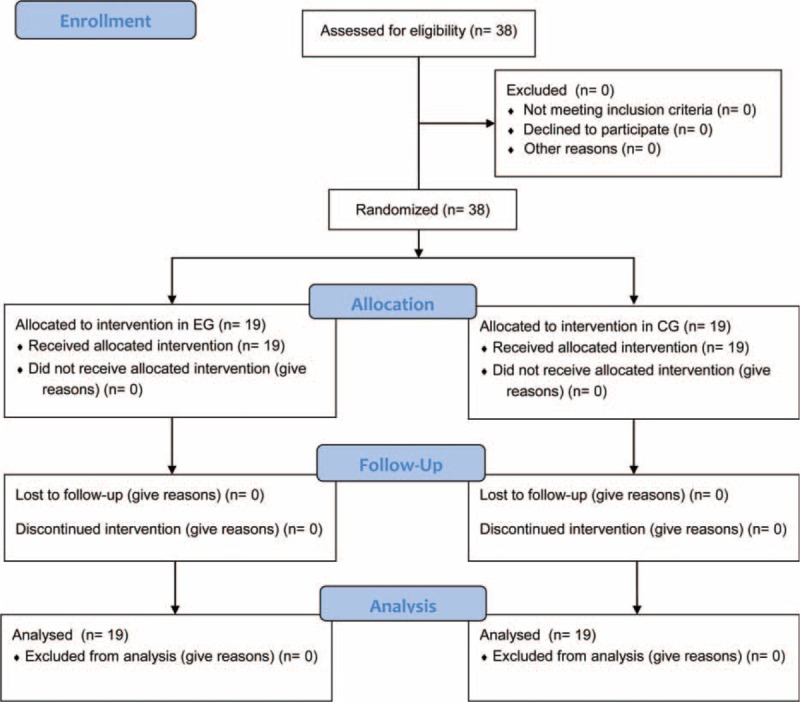
Flow of study.

Our RCT was carried out in compliance with the Declaration of Helsinki and the principles of the Italian data protection act (196/2003). Our trial and methods were approved by our institution's research ethics committee (Consiglio di Dipartimento SPPF Prot. No. 285/2015; punto all’ordine del giorno numero 12; 285–2015/MEDF-02/11). All participants were recruited from the University of Palermo Sports Center (CUS Palermo, Palermo, Italy). Prospective participants were individuals with a diagnosis of NSLBP who had experienced pain for >12 months. Individuals with a positive diagnosis of spinal stenosis, radiculopathy, any other specific cause of spinal dysfunction and pain, or serious underlying condition, such as cancer or infection, were excluded. Thirty-eight participants were enrolled into our RCT and allocated to 2 groups: the experimental group (EG) and the control group (CG) using a 1:1 randomization strategy. The allocation sequence was computer generated, with group allocation conducted by a research assistant who did not participate in any component of the study (Figure 1). The Consolidated Standards of Reporting Trials (CONSORT) Statement was set as a standard.^14^ Group characteristics are reported in Table 1. The groups were comparable in terms age and relevant anthropometric characteristics. Participants in the EG completed a Pilates matwork exercise program, without the use of nonsteroidal anti-inflammatory medications (NSAIDS). The Pilates matwork program is described in Table 2. Participants in the CG conducted were not actively monitored, continuing their own social activities and usual treatment, including the use of NSAIDs. The anthropometric characteristics of all participants were collected through a stadiometer (Seca 22 ± 1 mm approximation, Hamburg, Germany).

**TABLE 1:**
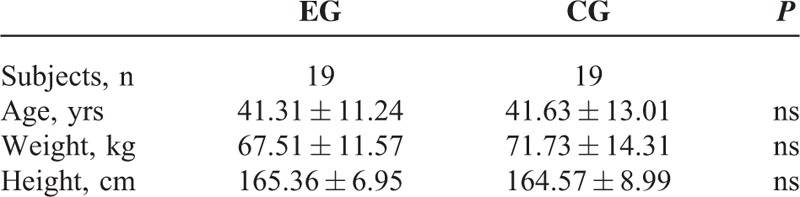
Participants’ Anthropometric Characteristics

**TABLE 2:**
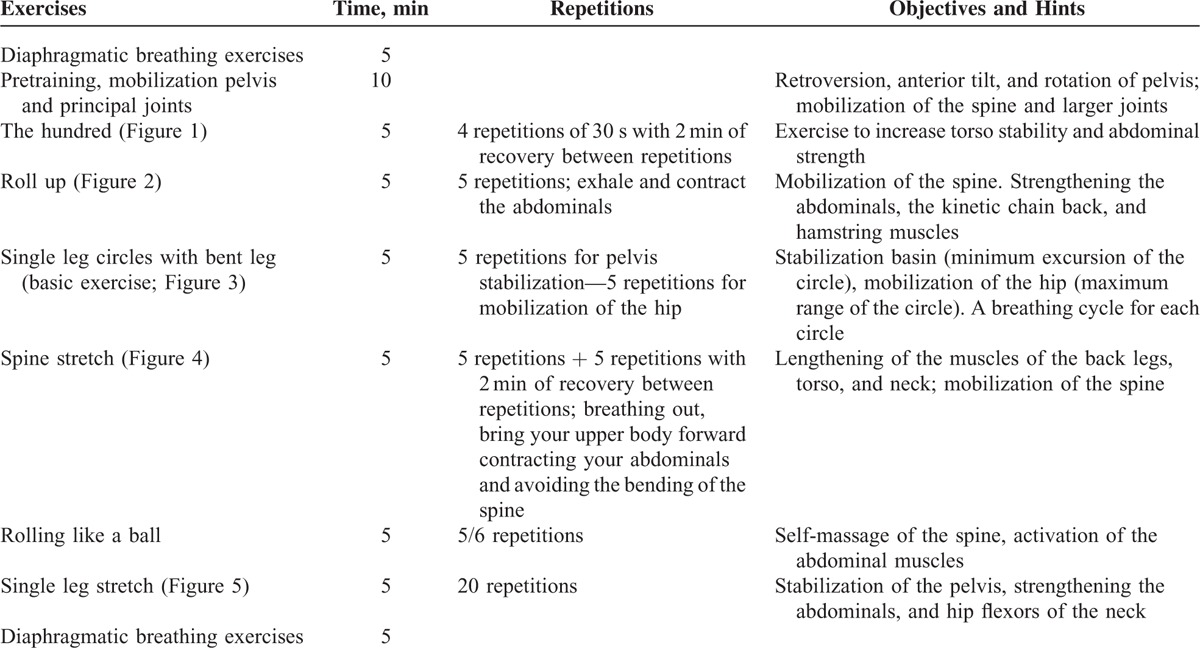
Pilates Matwork Designed by Authors

### Outcome Measurements

The Oswestry Disability Index (ODI) posturography assessments were administered by an examiner blinded to group assignment at the following times: T^0^, immediately prior to the study randomization (baseline) and T^1^, 14 weeks after T0 (conclusion of the Pilates program). The ODI provides a measure of an individual's pain and permanent functional disability related to their LBP. The test is considered to be the “Gold Standard” of low back functional outcome measures.^15^ The ODI comprises 10 items, with the score registered through an interview process. For each section, the total possible score is “5”; if the first statement is attributed a score of “5”, other statements in the section are scored as “0” and the LBP-related disability is considered to be minimal. In contrast, if the last statement is attributed a score of “5”, the LBP-related disability on that item is considered to be a maximum. The total possible score is 50, with a standardized formula used to transform to a percentage score of disability.^15^ For the posturography assessment, each participant performed the Romberg test using standardized positioning: feet placed side-by-side, forming an angle of 30° with both heels separated by 4 cm.^16^ Posturography was measured using the FreeMed posturography system, including the FreeMed baropodometric platform and FreeStep v.1.0.3 software. The system samples postural sway at 400 Hz, in real time. The sensors, coated with 24K gold, guarantee repeatability and reliability of the instrument (produced by Sensor Medica, Guidonia Montecelio, Roma, Italy). Participants were asked to hold the standardized Romberg test position on the baropodometric platform. Data from the platform were converted in accordance with instructions provided by the manufacturer and transformed into coordinates of the center of pressure (CoP). Participants repeated the static standing measures with eye open (OE) during the first analysis and with eyes close (CE) during the second analysis. The following parameters of the statokinesigram were considered for both CO and CE conditions: length of sway path of the CoP (SP); ellipse surface area (ES); coordinates of the CoP coordinates along the frontal (X; right-left; x-mean) and sagittal (Y; forward-backward; y-mean)^17^ planes.

### Intervention

**FIGURE 2:**
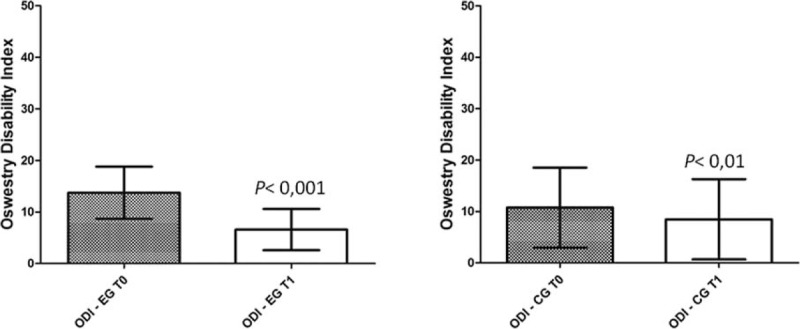
Scores obtained in the Oswestry Disability Index (score range from 0 up to 50).

**FIGURE 3:**
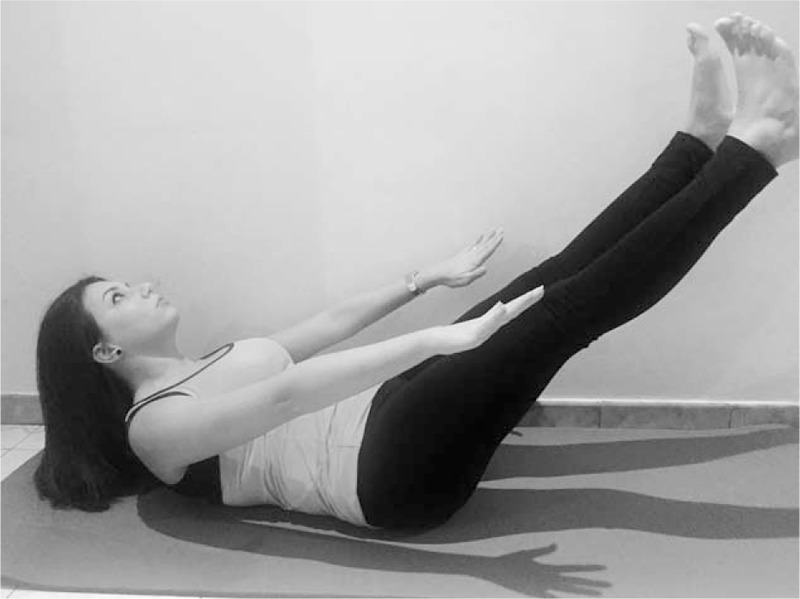
Pilates exercise - The Hundred.

**FIGURE 4:**
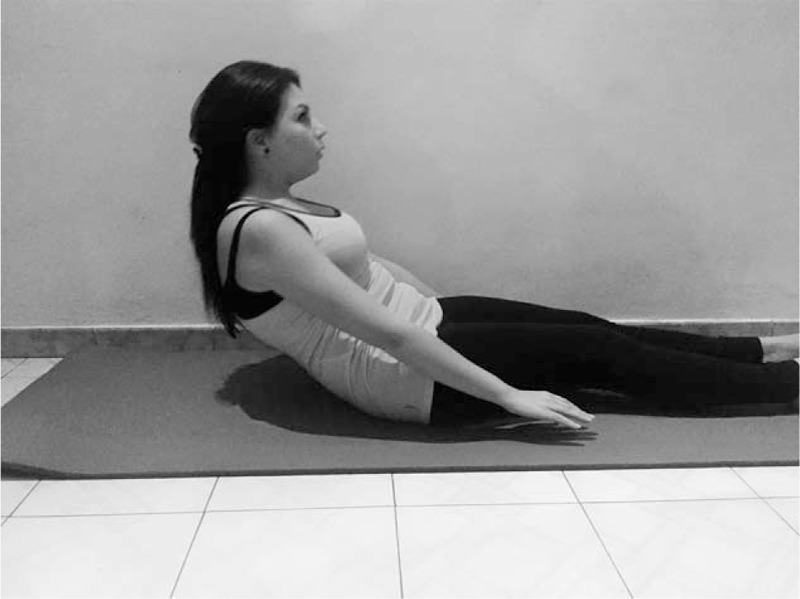
Pilates exercise - The Roll Up.

**FIGURE 5:**
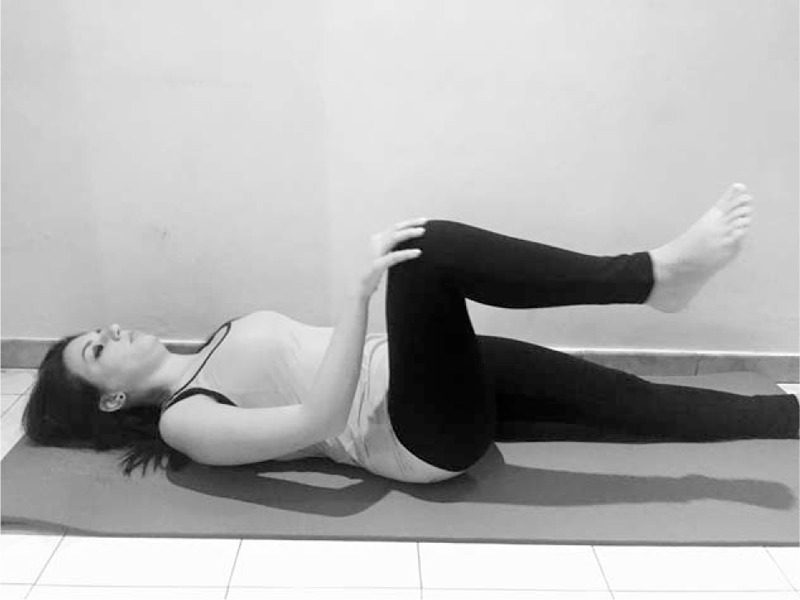
Pilates exercise - Single Leg Circles with bent leg.

**FIGURE 6:**
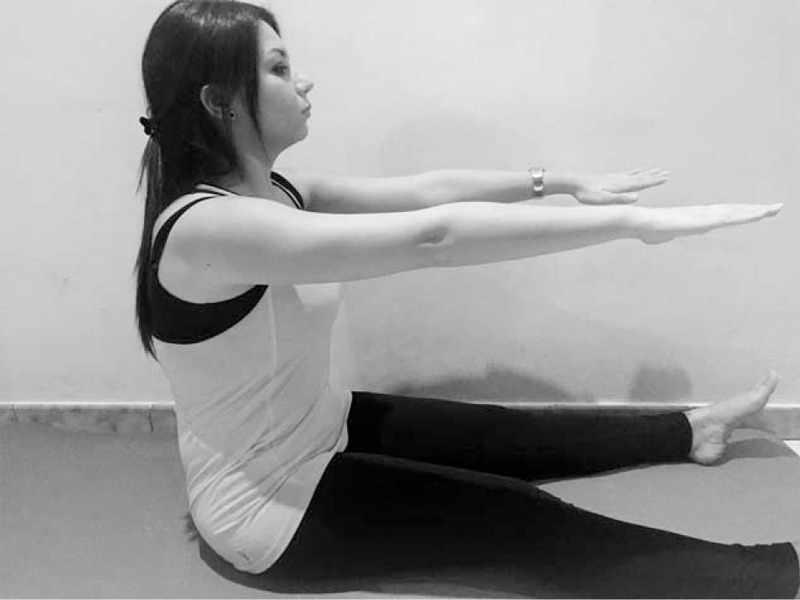
Pilates exercise - Spine Stretch.

All Pilates matwork sessions were conducted at the University Sports Center, Palermo (CUS Palermo) under the supervision of a sport and exercise science specialist with 10 years of experience in the Pilates method. Classes were 50 min in duration, following a pre-established Pilates protocol (Table 2). The EG was divided into 2 classes and the intervention was held 3 times per week. All exercises were completed at each session and could be performed at 2 levels of difficulty: basic and intermediate. Representative demonstrations of each exercise are shown in Figures 2–6: the hundred (Figure 2), roll up (Figure 3), single leg circles (Figure 4), spine stretch (Figure 5), rolling like a ball, and single leg stretch (Figure 6). All exercises were performed on a rubber mat of minimum 3/4 inch thick.

### Statistical Analysis

Statistical analysis was performed by 1 of the authors who is a statistician and epidemiologist. All data were coded on Excel file. Statistical analysis was performed using StatSoft's STATISTICA software (Windows, version 8.0; Tulsa, OK) and GraphPad Prism software (Windows, version 5.0; La Jolla, CA). Paired *t* test (*P* < 0.05) was used to detect significant differences in the posturography, before and after the Pilates’ intervention. Before and after differences in ODI were evaluated using Wilcoxon matched pairs test.

## RESULTS

**FIGURE 7:**
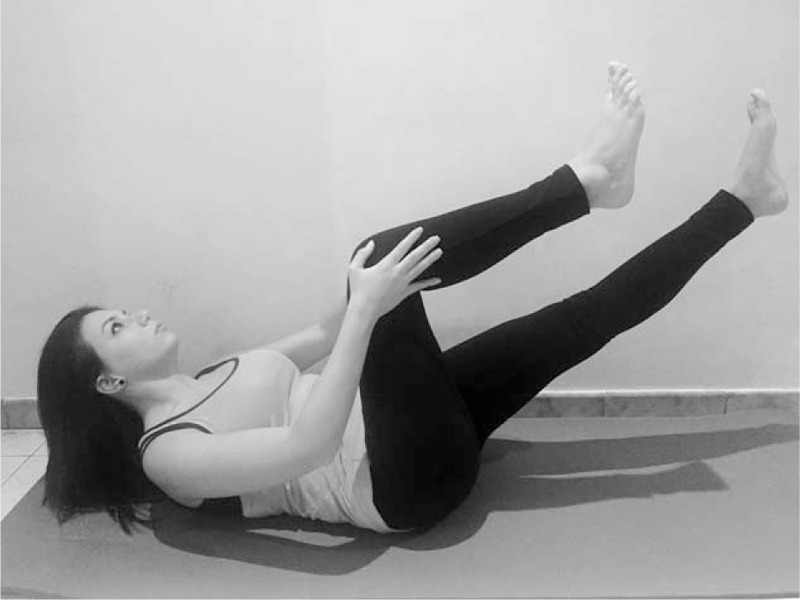
Pilates exercise - Single Leg Stretch.

The posturography results at T^0^ and T^1^ are reported for the EG (N = 19) and CG (N = 19) in for OE condition in Table 3 and CE in Table 4. There were no between-group differences in anthropometric variables at baseline, indicative of the homogeneity of the samples. There were significant before and after differences on all measured variables of posturography with both OE and CE in the EG (*P* < 0.05). Under the OE condition, there were significant decreases (paired *t* test) in SP (*P* < 0.001), ES (*P* < 0.05), and y-mean (*P* < 0.0001) after the Pilates exercise intervention. Results were similar under CE conditions, with significant decreases in SP (*P* < 0.0001), ES (*P* < 0.01), and y-mean (*P* < 0.0001) following the Pilates program. There was no statistical difference in posturography measurements between T^0^ and T^1^ in the CG. The ODI at T^0^ and T^1^ are reported in Figure 7. After our 14-week study protocol, there was a significant reduction in pain for both groups EG, T0, 13.7 ± 5 and T1, 6.5 ± 4 (*P* < 0.001) and CG, T0, 10.7 ± 7.8 and T1, 8.4 ± 7.8 (*P* < 0.01). The extent of reduction in ODI score was greater for the EG.

**TABLE 3:**
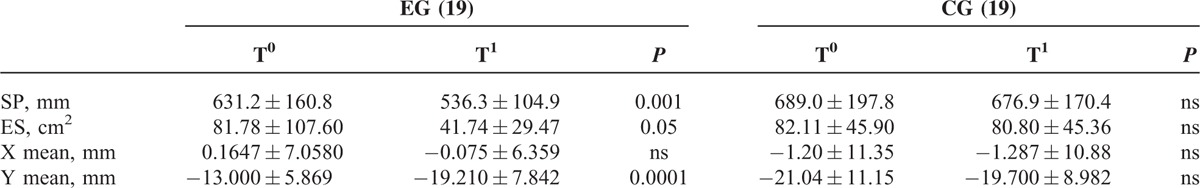
Scores Obtained in Posturography Analysis With Open Eyes

**TABLE 4:**
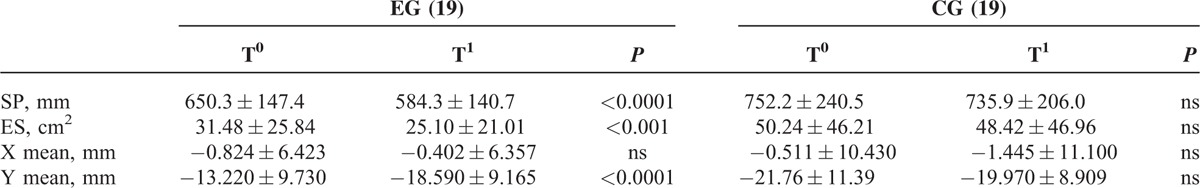
Scores Obtained in Posturography Analysis With the Closed Eyes

## DISCUSSION

The purpose of our RCT was to investigate the effectiveness of a Pilates exercise intervention on pain perception and balance control of people with chronic NSLBP. We provide evidence of a positive effect of Pilates exercises in improving posturography variables. A number of previous studies have evaluated the effects of Pilates on balance control,^18–20^ with recent systematic reviews reporting outcomes across studies to be inconsistent and, perhaps, even misleading.^21–23^ In our systematic review and critical appraisal of evidence, we concluded that Pilates exercises were more effective than no treatment or minimal physical exercise interventions in the management of NSLBP. However, the frequency and the intensity/workload of the Pilates protocols used to achieve these positive outcomes have been vague and often undefined.^6^ The Pilates program we used in our RCT incorporated both physical and mental elements, focusing on “the core”; with specific strengthening and control of abdominal, gluteal, and paraspinal muscles.^24^ In addition to incorporating the above-mentioned “core” objectives, we sought to create a definite Pilates training protocol, including specific volume and intensity; with easy to apply instructions to achieve a high rate of reproducibility. Our findings of effectiveness of our Pilates intervention in lowering ODI scores and improving posturography variables is in agreement with findings by da Fonseca et al who reported a reduction in LBP and increased vertical ground reaction force after 15 sessions of Pilates training.^25^ There is sufficient evidence regarding altered muscle recruitment patterns,^26,27^ inappropriate postural control^28^ and over-reliance on distal proprioception, due to impaired proprioception from proximal segments, in patients with NSLBP.^29^ Despite these findings, a recent systematic review provided no conclusive evidence to support the implementation of proprioceptive training interventions in the rehabilitation programs of patients with back- or neck-pain.^30^ However, in 2012, Paolucci et al^31^ did reported improvement in postural stability for trunk realignment and the control in patients with back pain using a proprioceptive rehabilitation program. Paolucci et al^31^ further demonstrated improvement in stability in patients with back pain following a specific program of exercise. Rogers and Gibson^32^ provided evidence of positive outcomes of an 8-week program of traditional Pilates mat exercises in improving body composition, muscle endurance, and flexibility. We confirm these findings, providing evidence that a 14-week program of Pilates exercise is sufficient to modify posturography in individuals with chronic NSLBP. In their 2012 study, Bird et al^33^ indicated an improvement in static and dynamic balance in elderly individuals after a 16-week Pilates program. They also reported lowering of pain perception, confirming previously reported benefits of Pilates on pain.^34–37^ Gladwell et al^38^ reported a 6-week program of Pilates exercise as being sufficient to improve general health, pain, flexibility, and proprioception in individuals with chronic LBP. In 2014, similarly, Natour et al^20^ demonstrated the added benefits of Pilates exercises in reducing pain in patients with LBP using NSAIDS. Our data in fact demonstrated the effectiveness of a Pilates intervention in achieving higher pain reductions than a group of individuals using NSAIDs. In conclusion, the Pilates exercise protocol we designed and implemented produced favorable outcomes in terms of pain in individuals with NSLBP compared to a CG with no exercises but NSAIDs use. Additionally, our 14-week program of Pilates exercise improved stabilometry in individuals with NSLBP. We propose that improvements in postural control specifically contributed in the greater extent in pain reduction in our exercise group, compared to no exercise CG. Although we cannot confirm this hypothesis with our current dataset, we do highlight the importance for further investigation of this hypothesis. Similar to outcomes by Sorosky et al,^39^ our outcomes are specific to individuals with LBP. Future research is needed to confirm the benefits of Pilates exercises for different LBP diagnoses, including lumbar radiculopathy and disc herniation. Our outcomes are also limited by our small study group (N = 38) and difficulty we found in recruiting a homogeneous group of people with similar LBP diagnosis. Despite these limitations, we support the feasibility of using posturography to evaluate postural instability in individuals with NSLBP. In fact, as there is no evidence of the sensitivity and specificity of routine plain radiographs in informing the clinical diagnosis of individuals with NSLBP, and improving outcomes, we recommend that posturography could provide be used, avoiding unnecessary exposure to ionizing radiation.^40,41^ Posturography could be included in routine diagnostic tests of individuals with NSLBP. In 2004, Segal et al^42^ reported the difficulty in evaluating postural deficits in this clinical population; posturography could be a solution to this problem. In the experimental and clinical research on therapeutic approaches for the management of LBP, there is differing views regarding the recommendation of exercise therapy, spinal manipulation, and muscle relaxants. Certainly, however, the literature indicates that early and gradual return to activities of individuals with LBP, and the discouragement of prescribed bed rest, is beneficial.^43,44^ Pilates exercises can be considered as spinal stabilization training.^45^ Critchley et al^46^ demonstrated an activation of the transversus abdominis and obliquus internus abdominis during Pilates exercises; however, this facilitation did not carry over into everyday postures. Unsgaard-Tondel et al^47^ analyzed the relationship between activation of deep abdominal muscles and NSLBP, providing evidence that improved strength of transversus abdominis was associated with clinically important long-term pain reduction. Moreover, Hodges and Richardson^27^ reported that the transversus abdominis was invariably the first muscle to be activated in many movements of daily living, and that delayed of contraction of transversus abdominis was indicative of deficits in motor control and inefficient muscular stabilization of the spine. It is possible that improvements in posturography after the Pilates program could be attributed to improved neuromuscular control of the transversus abdominals; further experimental research is required to confirm this mechanism of effectiveness. As mentioned previously, the LBP is 1 of the most common causes of disability, both physical and psychological.^1,2,48^ Moreover, the direct and indirect medical costs are in the range of more than $50 billion per annum, and could be as high as $100 billion at the extreme.^49^ If future studies could confirm our findings of the effectiveness of Pilates exercise in the management of LBP, this method could provide a low-cost strategy to counteract the increasing health and social burden of LBP and the associated rate of disability.
